# Green and Facile Synthesis of Pullulan-Stabilized Silver and Gold Nanoparticles for the Inhibition of Quorum Sensing

**DOI:** 10.1021/acsabm.1c00964

**Published:** 2022-02-03

**Authors:** Mohammadreza Ghaffarlou, Sedef İlk, Hamideh Hammamchi, Feyza Kıraç, Meltem Okan, Olgun Güven, Murat Barsbay

**Affiliations:** † Department of Chemistry, Hacettepe University, Beytepe, Ankara 06800, Turkey; ‡ Faculty of Medicine, Department of Immunology, Niǧde Ömer Halisdemir University, Niǧde 51240, Turkey; § School of Engineering Sciences in Chemistry, Biotechnology and Health, Department of Chemistry, Division of Glycoscience, KTH Royal Institute of Technology, Stockholm SE-10691, Sweden; ⊥ Department of Biology, Hacettepe University, Beytepe, Ankara 06800, Turkey; || Department of Micro and Nanotechnology, Middle East Technical University, Ankara 06800, Turkey

**Keywords:** pullulan, quorum sensing inhibition activity, antimicrobial activity, Ag and Au nanoparticles, supramolecular self-assembly

## Abstract

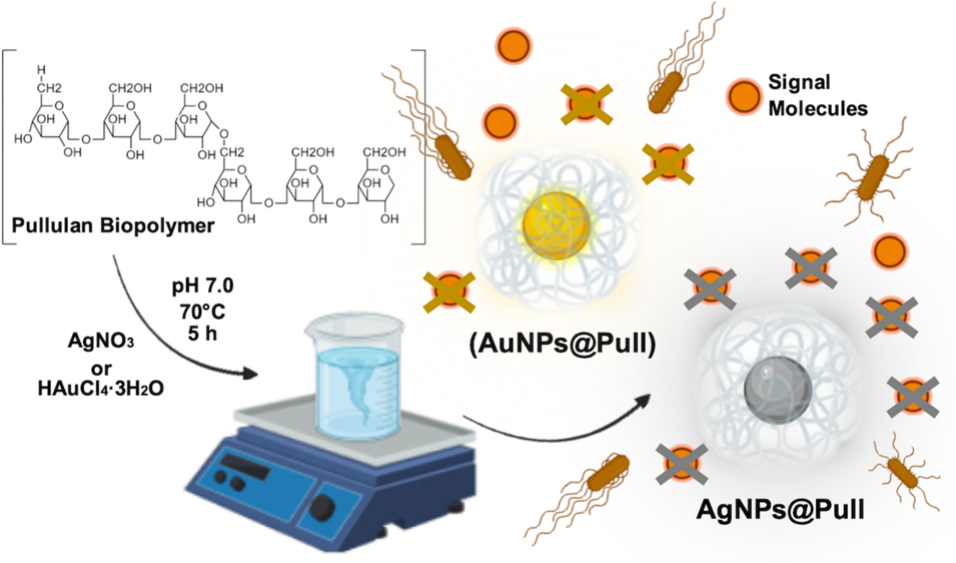

Pullulan (Pull) decorated with monodisperse Ag and Au nanoparticles (NPs) was synthesized by a simple and green method. Samples were characterized by FTIR, UV–vis, NMR, XRD, TGA, SEM, XPS, DLS, and TEM. SEM images showed highly oriented microforms reported for the first time for Pull, because of the supramolecular self-assembling behavior of Pull chains. Antimicrobial and quorum sensing (QS) inhibition activities were tested against six pathogen bacteria and reporter and biomonitor strain. Pull decorated with NPs, in particular, Ag-modified ones, outperformed pristine Pull. The cell proliferation was tested with an MTT assay. NPs-decorated Pull was studied for the first time as an inhibitory agent against bacterial signal molecules and found to be a good candidate. The promising performance of AgNPs@Pull compared to the commercial antibiotic gentamicin showed that it has great potential as a therapeutic approach to overcome the bacterial resistance that has developed against conventional antibiotics.

## 1 Introduction

Communication within bacteria is achieved through signal molecules. Bacteria can measure the density of the signaling molecules they produce, and therefore can sense the amount of other microorganisms around them.^1^ “Quorum sensing” (QS) is the way bacteria exhibit a variety of behaviors by detecting the signal molecules at a certain intensity. Gram-positive bacteria produce small oligopeptides, whereas Gram-negative bacteria produce acyl homoserine lactones.^2^ These small molecules, called autoinducers (AIs), stimulate the expression of several genes that lead to many bacterial functions such as virulence factor production, bioluminescence, biofilm differentiation, biosurfactant production, swarming, etc., when a high population density is reached.^3^ It is reported that if communication is interrupted by blocking the QS system, the bacteria cannot move in a coordinated manner and their pathogenic effects can be eliminated.^4,5^ Thus, inhibition of the QS system can lead to the development of a new generation of antimicrobials that allow the pathogenic properties of bacteria to be controlled without killing them.

Recently, there has been a great increase in the production of anti-QS compounds derived from plants and/or synthetic drugs. Compared to conventional antibiotics, these anti-QS agents have promising results in in vitro studies, but clinical uses are restricted because of obstacles such as instability, insolubility, low bioavailability, etc.^6^ For a better inhibition of the QS systems clinically, researchers are striving for nanoantimicrobial agents because o their higher solubility, effective delivery properties, and better penetration ability.

Silver nanoparticles (AgNPs) are attracting great attention in the development of new-generation anti-QS agents because of their excellent broad-spectrum antimicrobial activity.^7,8^ The promising performance of gold nanoparticles (AuNPs) in the QS inhibition is drawing attention as well among metal NPs.^9,10^ Extensively stable forms of these NPs are prepared by chemical reduction in aqueous or organic solvents;^11,12^ however, use of toxic reducing agents, insufficient performance of stabilizers, and generation of chemical byproducts are the limitations of this technique. Recently, green synthesis methods have been preferred over conventional methods using chemical agents in the production and stabilization of Ag and Au NPs.^13^ In this sense, natural, sustainable, and nontoxic polysaccharides are under extensive research for the green synthesis of Ag and Au NPs because of their structural diversity, renewability, and environmental friendliness. Therefore, various types of polysaccharides such as chitosan,^14^ starch,^15^ and pullulan^16^ have been used as reducing/stabilizing agents in the green synthesis of Ag and Au NPs.^17,18^

In this study, a simple and green method was employed for the synthesis of stable Ag and Au nanoparticles with monodisperse size distribution using pullulan as both the reducing and stabilizing agent. Following the chemical, structural, and thermal characterizations of NP-decorated Pull, the potential for use of AgNPs@Pull and AuNPs@Pull as inhibitory agents against bacterial signal molecules was studied for the first time. The findings showed that these hybrid polysaccharides constituted a strong alternative to commercial antibiotics as a modern therapeutic tool against bacterial resistance.

## 2 Materials and Methods

### 2.1 Materials

*A. pullulans* 201253 was obtained from the American Type Culture Collection and was maintained at 4 °C on Sabouraud dextrose agar (SDA) and subcultured regularly every 2 weeks. For long-term storage, cultures were maintained at −80 °C in a 15% glycerol solution. For inoculum preparation, *A. pullulans* was grown at 28 °C for 48 h in a medium shown in Table S1. Silver nitrate (AgNO_3_, ≥ 99.0%) and gold(III) chloride trihydrate (HAuCl_4_·3H_2_O, ≥ 99.9%) were purchased from Sigma-Aldrich. Luria–Bertani broth, Muller–Hinton agar, and other chemicals used in the antimicrobial analyses (Table S1) were purchased from Sigma-Aldrich. Mica was supplied from Agar Scientific. Twenty-five micrometer thick poly(ethylene-*alt*-tetrafluoroethylene) (ETFE) film (Tefzel 100LZ) was kindly donated by the Paul Scherrer Institute, PSI, Switzerland. Poly(styrene sulfonic acid)-grafted ETFE film (ETFE-*g*-PSSA, degree of grafting: 54%) was synthesized in a previous study of ours.^19^

### 2.2 Synthesis of Pull, AgNPs@Pull and AuNPs@Pull

First, microorganisms produced in SDA were inoculated into the prepared culture medium and incubated for 48 h at 28 °C. At the end of the incubation period, 5% (v/v) of microorganisms were transferred to the synthetic production medium in aseptic conditions and incubated at 28 °C, 200 rpm for 48 h. Next, the culture was centrifuged at 10 000 × *g* for 10 min to remove the microorganism. The biomass (mycelia and yeast-like cells) dry weight (BDW) was determined by washing the sediment by centrifuging with twice distilled water and drying at 80 °C overnight. To precipitate the exopolysaccharide, we transferred 15.0 mL of the supernatant into a test tube and added ethanol to the obtained solution at a ratio of 2:1 (v/v), and the resulting mixture was kept in a refrigerator at 4 °C for 12 h. The supernatant was subsequently separated by centrifuging (10 000 × *g*, 10 min) and was dried at 80 °C. Eventually, the final weight determined as that of exopolysaccharide. All measurements were done in triplicates.

Three milligrams of Pull was dissolved completely in 15.0 mL of deionized water under stirring at 40 °C. Then, 1.0 mL of 0.01 mg/mL silver nitrate (AgNO_3_) or 1.0 mL of 0.01 mg/mL HAuCl_4_·3H_2_O was added to this solution. The mixture was kept under continuous stirring for 5 h at room temperature. The metal ion-absorbed pullulan solution was heated to 70 °C and shaken for 5 h at 150 rpm to reduce Ag(I) or Au(III) into zerovalent metallic forms and eventually to obtain AgNPs@Pull and AuNPs@Pull. After heating, the color of Pull-Ag and Pull-Au solutions turned into light brown and pink, respectively, indicating the formation of metallic Ag and Au NPs. Solutions containing AgNPs@Pull and AuNPs@Pull were used without any purification. For analyses requiring solid samples, the solutions were freeze-dried.

### 2.3 Characterization of Pull, AgNPs@Pull, and AuNPs@Pull

Chemical characterizations of pristine and hybrid pullulan samples were carried out by UV–vis, ATR-FTIR, X-ray photoelectron, and NMR spectroscopy. For the UV–vis analysis, Varian Cary100 spectrophotometer was used. The spectra were recorded between 200 and 800 nm in a 1.0 mL quartz cuvette at room temperature. For the ATR-FTIR analysis, IR spectra were recorded at ATR mode on a PerkinElmer Spectrum One model spectrometer. The spectra were obtained by 32 scans with 4 cm^–1^ resolution at a scan range from 4000 to 400 cm^–1^. For the NMR analysis Bruker 400 Ultra Shield spectrometer was operated at 400 MHz and 25 °C after samples were dissolved in deuterium oxide, D_2_O (acetone as internal standard, ^1^H: (CH_3_)_2_CO at δ = 2.2 ppm; ^13^C: (CH_3_)_2_CO at δ = 30.2 ppm). A Thermo Scientific high-performance surface analysis instrument with a monochromatized Al–Kα X-ray source (1486.6 eV) was used to determine surface elemental compositions by XPS. The anode current was 20 mA and pressure in the chamber was maintained at 3 × 10^–8^ mBar or lower.

X-ray diffraction (XRD) analyses were carried out with a PANalytical X’Pert powder diffractometer using CuKα radiation in the range of 2θ = 10–90° and at 8 rpm rotation for homogeneous data collection. The thermal stabilities were investigated by TGA using a PerkinElmer Pyris model thermogravimetric analyzer from room temperature to 850 °C under a N_2_ atmosphere at a scanning rate of 10 °C/min.

Hydrodynamic diameters, size distributions, and surface charges in aqueous solution were measured by dynamic light scattering (DLS) using a Malvern Nano ZS Zetasizer. All DLS measurements were repeated at least four times using the same sample. Freshly prepared samples were also tested and results were consistent. For scanning electron microscopy (SEM) imaging, samples were prepared in deionized water at a concentration of 0.1 mg/mL, and each solution was individually dropped onto the substrate surface and allowed to dry slowly at room temperature. Before each SEM measurement, a mica flake was removed from the surface with the help of an adhesive tape to obtain a clean mica layer free of any contamination. Other surfaces were cleaned in ethanol and acetone before SEM analysis and dried in a vacuum. An FEI Quanta 200FEG microscope was used to take the SEM images. The substrate surfaces were coated with platinum (ca. 5 nm) before measurement. An FEI Tecnai G2 F30 transmission electron microscope (TEM) operated at 100 keV was used for imaging of Ag and Au NPs.

### 2.4 Bacterial Assays of Pristine and NP-Decorated Pull Samples

Bacterial assays of as-synthesized AgNPs@Pull and AuNPs@Pull and pristine Pull include testing of antimicrobial and QS inhibition activities as well as cell proliferation studies as described below.

#### 2.4.1 Antimicrobial Activity of Pull, AgNPs@Pull, and AuNPs@Pull

*Escherichia coli* (*Ec*) ATCC 25922, *Staphylococcus aureus* (*Sa*) ATCC 25923, *Streptococcus mutans* (*Sm*) ATCC 25175, *Salmonella enterica serotype typhmurium* (*St*) SL 1344, *Bacillus thuringiensis* (*Bt*) and *Pseudomonas aeruginosa* (*Pa*) ATCC 27853 were used for antimicrobial tests of AgNPs@Pull and AuNPs@Pull and pristine Pull (each 0.2 mg/mL). Microorganisms were subcultured on Luria–Bertani (LB) agar at 37 °C for 24 h. Antimicrobial activities of pristine and NP-decorated Pull were determined by the disc diffusion test according to the modified standard method (Clinical and Laboratory Standards Institute (CLSI), Performance Standards for Antimicrobial Disk Susceptibility Tests, 2012). The suspension of microorganism was adjusted to 0.5 McFarland as the reference standard. The bacteria culture (100 μL) was swabbed (1 × 10^6^ cells/mL) onto Muller–Hinton agar on a Petri plate and filter discs (6 mm in diameter) were placed on the inoculated agar. Pristine and NP-decorated Pull samples were loaded on the discs (20 μL) and the tested Petri plates were incubated overnight at 37 °C. The discs loaded with gentamicin (20 μL) and solvent only were used as positive and negative controls, respectively. All tests were repeated three times. The clear zones (no bacterial growth) around discs were measured as inhibition zones. The results were demonstrated as the mean diameter of the inhibition zone in mm ± standard deviation (mean ± SD).

#### 2.4.2 QS Inhibition Activity of Pull, AgNPs@Pull, and AuNPs@Pull

The QS inhibition properties of pristine Pull, AgNPs@Pull, and AuNPs@Pull were determined against a reporter strain *Chromobacterium violaceum* (*C. violaceum*) CV026 and a biomonitor strain *C. violaceum* ATCC 12472 by disc diffusion tests^20^ at concentrations of 0.2, 0.1, 0.05, and 0.025 mg/mL. The suspension of bacteria was first subcultured in LB broth at 30 °C for 24 h. Petri plates loaded with LB soft agar were then prepared and signal molecules (C6-HSL, 0.25 μg/mL) were added. The strain *C. violaceum* CV026 swabbed onto the plates and sterile discs (6 mm in diameter) were replaced onto an agar plate. Twenty microliters of each Pull sample was loaded on the disc and plates were incubated at 30 °C for 24 h. Gentamicin solution (10 μg/mL) and solvent-only media were used as positive and negative controls, respectively. Halo formation with a purple background around the discs was accepted as the QS inhibition zones of each sample. All tests were repeated three times. The results were demonstrated as the mean diameter of the inhibition zone in mm ± standard deviation (mean ± SD).

To determine the quantitative violacein inhibition of pristine Pull, AgNPs@Pull, and AuNPs@Pull with different concentrations (0.2, 0.1, 0.05, and 0.025 mg/mL), *C. violaceum* ATCC 12472 was used as the bacterial culture according to the method by Ilk et al.^20^ First, the suspension of each sample (1.0 mL) was poured into the *C. violaceum* ATCC12472 cultures. Gentamicin solution (10 μg/mL) and solvent-only media were used as positive and negative controls, respectively. After 24 h of incubation, the culture of each sample was centrifuged (11000 rpm, 10 min) to precipitate the insoluble violacein and bacterial cells and the absorbance of supernatant was measured at 585 nm using UV–vis spectrophotometer (Schimadzu, UV1800). The assay was repeated three times. The results were demonstrated as the mean diameter of the inhibition zone in mm ± standard deviation (mean ± SD). The violacein inhibition was expressed as percentage. The percent violacein inhibitions of pristine Pull, AgNPs@Pull, and AuNPs@Pull were evaluated by the following formula:
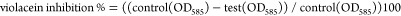


#### 2.4.3 Cell Culture and Proliferation Studies

The effect of AgNPs@Pull and AuNPs@Pull on L929 cell adhesion was determined with cell suspension incubated with different concentrations of NP-decorated hybrid polysaccharide. The MTT (3-(4,5-dimethylthiazol-2-yl)-2,5-diphenyltetrazolium bromide) assay was applied as a simple and nonradioactive colorimetric method to measure cell cytotoxicity, proliferation, or viability. To determine cell viability/cytotoxicity, we cultured L929 cells at a density of 1 × 10^4^ cells/well in DMEM supplemented with 10% FBS and 100 mg/mL penicillin/streptomycin at 37 °C in 5% CO_2_ atmosphere. The medium in the wells was then replaced with fresh medium containing varying concentrations of AgNPs@Pull and AuNPs@Pull (50, 100, 150 μg/mL). The tests were also performed in the absence of AgNPs@Pull and AuNPs@Pull. After 24, 48, and 72 h of incubation, 20 μL of MTT dye solution (5 mg/mL in phosphate buffer at pH 7.4) was added to each well. After 4 h of incubation at 37 °C and 5% CO_2_ for exponentially growing cells and an additional 15 min for steady-state confluent cells, the medium was removed and formazan crystals were solubilized with 200 μL of DMSO. The absorbance of each well was then read on a microplate reader at 550 nm. The spectrophotometer was calibrated to zero absorbance using culture medium without cells. The relative cell viability (%) of control wells containing cell culture medium without NP-decorated Pull was calculated using the following formula:
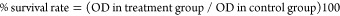
All samples were run in six replicates and the experiments were repeated twice.

#### 2.4.4 Statistical Analysis

Statistical analysis to compare antimicrobial inhibition zone values (mm) against Gram-positive and Gram-negative bacteria and cytotoxicity tests of different concentrations by means of time dependent activities against L929 cell line of pristine Pull, AgNPs@Pull and AuNPs@Pull was performed using “IBM SPSS Statistics ANOVA” program. The significance level was accepted as *p* < 0.05.

## 3 Results and Discussion

### 3.1 Synthesis of Pristine Pull, AgNPs@Pull, and AuNPs@Pull

**Figure 1:**
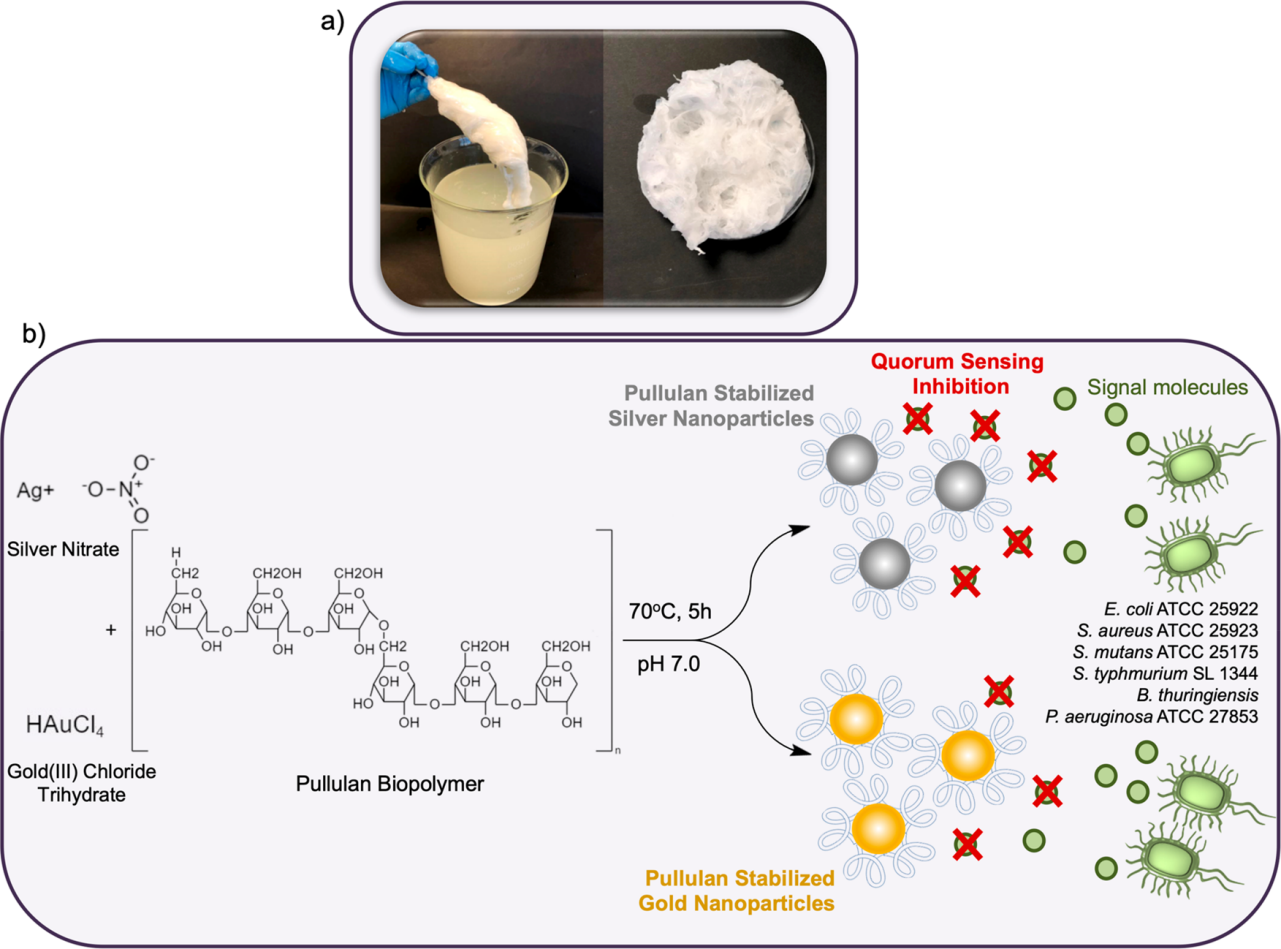
(a) Digital image of Pullulan biopolymer from *A. pullulans* 201253. (b) Schematic representation of AgNPs@Pull and AuNPs@Pull formation and their QS inhibition.

The maximum Pull production was accomplished under the conditions specified in Table S2. The high efficiency production of 32.89 g/L Pull obtained from *A. Pullulans* 201253 in the optimized medium is shown in Figure 1a. Pull production by *A. Pullulans* 201253 continued to increase until it reached its maximum amount of 8.24 g/L at 28 °C. The optimal time for this production was determined as 3 days. All measurements were done in triplicates.

Ag and Au NPs were obtained by the reduction of Ag(I) and Au(III) ions adsorbed by Pull to zerovalent metallic NPs simply by heating (70 °C). Pull behaves as both the reducing and the stabilizing agent through its functional groups. The hydroxyl groups on the Pull chains are oxidized to carboxylate while Au^3+^ is reduced to Au^0^ and similarly Ag^+^ to Ag^0^, forming the AuNPs@Pull and AgNPs@Pull, respectively. When a certain concentration of reduced atoms is reached, they constitute clusters behaving as nucleation centers, which further lead to the formation of larger aggregates. This aggregation process forms larger particles. Once these large particles interact with the polymer matrix, coalescing stops.^21^ The hydroxyl groups on the Pull chains are also effective in coating NPs, and the steric Pull stabilizing matrix with Au and Ag NPs is attained through hydrogen bonds.^18^ Metal NPs are well-known to be stabilized by polymer chains that interact strongly with them.^22,23^ With its dense functional groups interacting with Ag and Au NPs, Pull acts as a stabilizer that promotes the formation of Ag and Au NPs and prolongs their stability by preventing their aggregation. The reducing and stabilizing effect of Pull on the formation of Ag and Au NPs was schematically represented in Figure 1b.

### 3.2 Characterization of Pull-Based Samples

**Figure 2:**
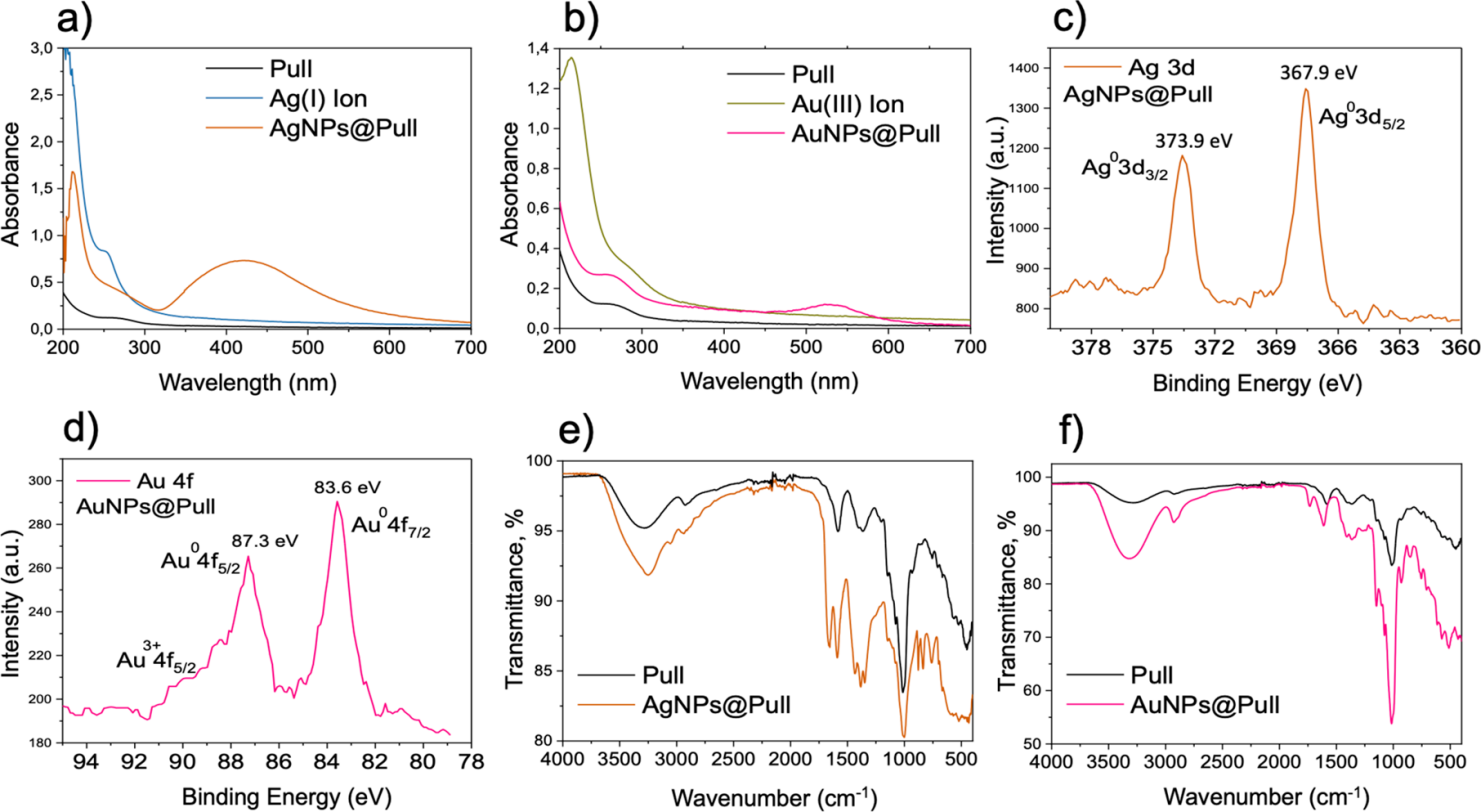
UV–vis absorption spectra of (a) pristine Pull, Ag(I)-Pull solution before reduction, and AgNPs@Pull, and (b) pristine Pull, Au(III)-Pull solution before reduction, and AuNPs@Pull. (c) High-resolution Ag 3d XPS spectrum of AgNPs@Pull. (d) High-resolution Au 4f XPS spectrum of AuNPs@Pull. FTIR spectra of (e) pristine Pull and AgNPs@Pull and (f) pristine Pull and AuNPs@Pull.

The formation of Ag and Au NPs was primarily carried out with UV–vis spectroscopy. AgNPs@Pull formed a broad absorption band in the wavelength range of 380–450 nm due to the surface plasmon resonance (SPR) transition confirming the production of AgNPs (Figure 2a).^16^ For the case of AuNPs@Pull, a localized SPR peak in the wavelength range of 520–550 nm was observed, similarly proving the establishment of AuNPs (Figure 2b). The existence of interactions between Pull and both metal ions was proved via the shifts in the UV bands, which are in good agreement with the literature.^24,25^ The shift in AgNPs@Pull was found to be greater than that of AuNPs@Pull, indicating a relatively stronger interaction. After the formation of NPs, characteristic metallic zerovalent NP peaks emerged, yet some ionic forms remained. The ionic peak intensity decreases dramatically after the reduction. The formation of Ag and Au NPs was also confirmed by XPS analysis. The wide-scan XPS spectra presented in Figure S1 clearly show the incorporation of Ag and Au into the major elements (C and O) that make up the pullulan structure. The high-resolution Ag 3d peaks for the as-prepared sample are located at 367.9 eV (3d_5/2_) and 373.9.0 eV (3d_3/2_), with a spin–orbit splitting of 6.0 eV (Figure 2c). This is attributed to zerovalent metallic Ag,^26,27^ consistent with the XRD result presented below. The core-level XPS spectrum of Au 4f, shown in Figure 2d, can be characterized by three pairs of peaks. The peaks at 87.3 and 83.6 eV correspond to Au 4f_5/2_ and Au 4f_7/2_ spin–orbit doublets of elemental gold (Au^0^).^28,29^ Another peak is seen around 90.4 eV, corresponding to the +3 oxidation state of Au,^29^ indicating that the reduction is not complete and some gold ions remain in the structure.

The FTIR spectrum of purified Pull shows signals at 3310 and 2925 cm^–1^ corresponding to O–H and C–H stretching vibrations of Pull (Figure 2e, ,f, in black), respectively, in agreement with previous data.^30^ The characteristic absorption bands at 1373 and 1014 cm^–1^ are assigned to vibrations of C–O–H and C–O stretching, respectively. The presence of α-configuration is confirmed by the peak at around 850 cm^–1^. The signal at 928 cm^–1^ corresponds to α-1,6-d-glycosidic, whereas the peak at 752 cm^–1^ is assigned to α-1,4-d-glycosidic bands. These results are consistent with the characteristic absorption bands of Pull.^18,31^ After the reduction, new peaks emerged and the intensities of some existing peaks changed. In the spectra of the NP-decorated Pull, the peaks appearing around 1416–1423 cm^–1^ and 880–920 cm^–1^ are attributed to the symmetric stretching of dissociated carboxyl groups and C–O–C, respectively. The intensity of the COO^–^ peaks increased at 1383 and 1583 cm^–1^ (for AgNPs@Pull), and 1363 and 1602 cm^–1^ (for AuNPs@Pull). These spectral changes prove reduction of metal ions and oxidation of Pull. The intensity of the carbonyl peak for AgNPs@Pull was found to be higher compared to that of AuNPs@Pull, which can be attributed to a more effective reduction in the case of silver ion. This finding may be important in explaining the high QS performance of AgNPs@Pull, which will be discussed later.

The zeta potential data measured by DLS showed that the surface charges of both AgNPs@Pull and AuNPs@Pull were more negative compared to pristine Pull. The zeta potential of pure Pull solution was determined as −2.98 ± 4.31 mV, whereas the charges were −23.2 ± 4.85 mV and −17.9 ± 6.92 mV for AgNPs@Pull and AuNPs@Pull solutions, respectively. On the basis of this data, it is concluded that Ag and Au ions are reduced to their metallic forms, whereas the hydroxyl groups of Pull are oxidized to carboxylate, which explains the measured negative surface charge. A typical size distribution of Pull is shown in Figure S2a. In nanosized region, two peaks are prominent around 39 and 290 nm. There is another peak at around 5 μm. These microsized aggregates are commonly observed in aqueous polysaccharide solutions.^32,33^ The peak at ∼290 nm can be ascribed to the aggregates of the isolated smaller nanoparticles observed at ∼39 nm. The distributions of AgNPs@Pull and AuNPs@Pull do not exhibit the micrometer-sized peak as shown in Figure S2a, b. The diameters of the isolated nanoparticles (∼40 nm or less) and their aggregates (>100 nm) are smaller compared to pristine Pull, indicating that the aggregates evolve to become smaller after the coordination of Ag and Au NPs. After 6 months of storage at ambient temperature, the size distributions of the aqueous solutions of AgNPs@Pull and AuNPs@Pull remained nearly the same with no accompanying changes in appearance.

The ^13^C NMR spectrum of pristine Pull (Figure S3a) gives characteristic signals for this polysaccharide (C1: 98.0–100.1–100.7 ppm, C4: 69.9 ppm and C2,3,5: 72.1 to 77.1 ppm, and C6: 60.1–60.4–66.2 ppm).^34,35^ The ^1^H NMR spectrum (Figure S2a) also confirms pullulan structure with the corresponding peak assignments at 4.84 ppm for H-1, 3.51 to 3.86 for H-2–H-5, and 3.83 and 3.91 for H-6 hydrogens.^36^ With the addition of AgNPs, the above peak assignments shifted at C1: 97.9–99.7–100.2 ppm, C4: 69.4 ppm and C2,3,5: 70.3 to 73.4 ppm, and C6: 60.3–60.6 ppm in the ^13^C NMR spectra (Figure S3b). The ^1^H NMR spectrum (Figure S4b) shows changes in the AgNPs@Pull structure from 4.84 to 3.36 ppm for H1–H6 hydrogens with slight shifts and intensity changes in signals. The AuNPs@Pull ^13^C NMR and ^1^H NMR spectra (Figures S3c and S4c) also confirmed structural assignments between 97.8 and 99.7–100.1 ppm for C1 and 60.3–60.6 ppm for C6 carbon atoms and left-shifted assignments between 4.85 to 3.37 for H1–H6 protons.

**Figure 3:**
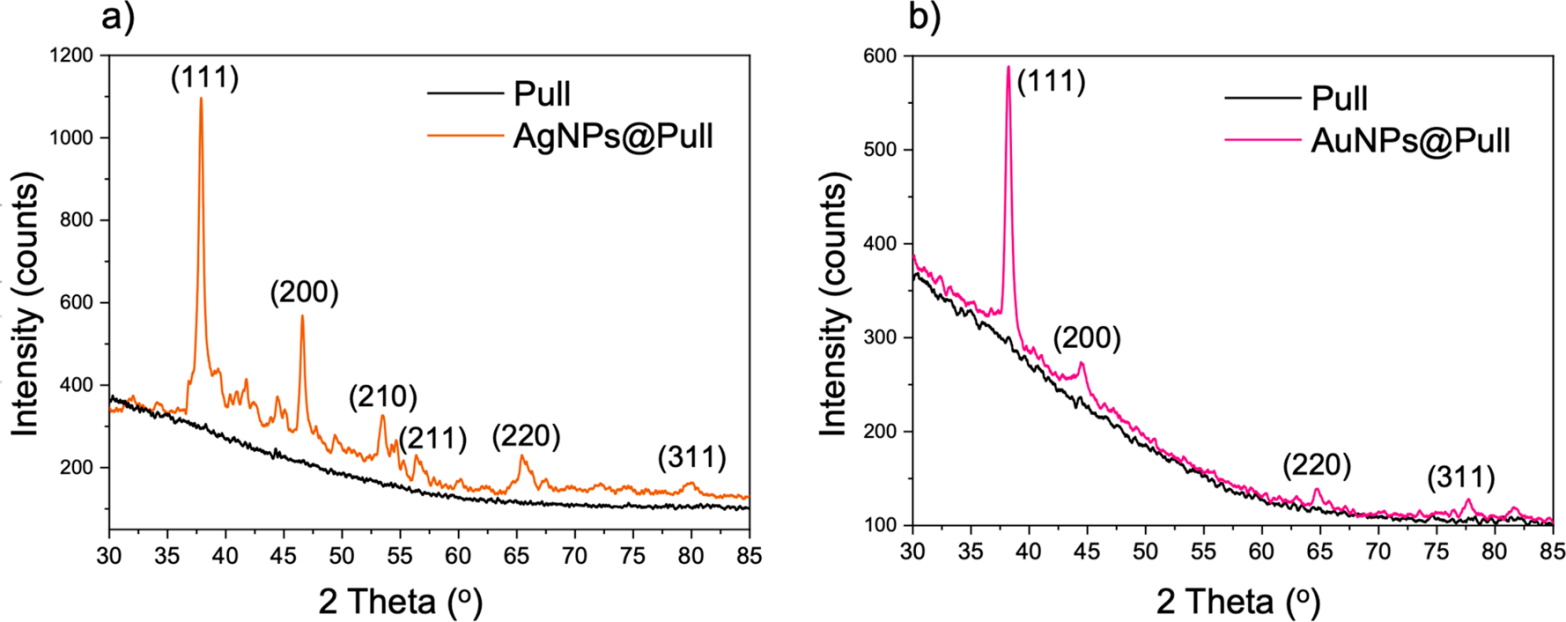
Comparison of XRD pattern of pristine pullulan with (a) AgNPs@Pull (b) AuNPs@Pull.

The XRD analysis yields the status of the elemental metals, in our case, Ag and Au involved in the formation of nanostructures.^24,37^ Diffraction patterns from 30 to 85° 2θ were recorded for this purpose. In the case of AgNPs@Pull, six main peaks at 2θ values of 38.0, 46.6, 53.4, 56.3, 65.7, and 79.8° were detected that correspond to the (111), (200), (210), (211), (220), and (311) planes of metallic silver nanoparticles, respectively (Figure 3a). The resulting planes confirm the existence of the face-centered cubic (FCC) structure of crystalline AgNPs, are in good agreement with the literature.^16,24,38^ The (111) plane was found to be more intense compared to others, which was previously reported by Kanmani et al. as well and explained by the predominant orientation of the very plane. It was stated by Ganduri et al. that the high intensity peak for FCC materials is the (200) reflection, which was observed clearly in our spectrum. Some small unidentified peaks are also observed, which might have arisen from crystallization of the reducing/stabilizing agent Pull. This is in line with the discussions obtained by SEM, which will be presented later. Similar findings were mentioned in the literature.^16,24^ In the case of AuNPs@Pull, 2θ values of 38.2, 44.5, 64.7, and 77.7° were recorded, corresponding to the (111), (200), (220), and (311) planes of the FCC lattice, respectively, similar to the values for AgNPs (Figure 3b). The intense diffraction peak at 38.2° indicates that the preferred growth orientation of Au^0^ was fixed in the (111) direction.^39^ Overall, the spectrum represents the formation of Au nanocrystals.

TGA thermograms of pristine and NP-decorated Pull are given in Figure S5. The first mass loss in the TGA curve of Pull at around 100 °C is associated with the evaporation of adsorbed water molecules.^40^ A mass loss of about 55% (w/w) due to degradation of saccharide rings started at around 275 °C and ended at ∼400 °C.^41^ The third-stage mass loss at around 470 °C was due to byproduct formation of Pull.^42^ The thermal stabilities of both AgNPs@Pull and AuNPs@Pull were higher than that of Pull because of interactions of Pull and metal nanoparticles, leading to a higher chain compactness^43^ and an increase in the crystallinity of Pull.

**Figure 4:**
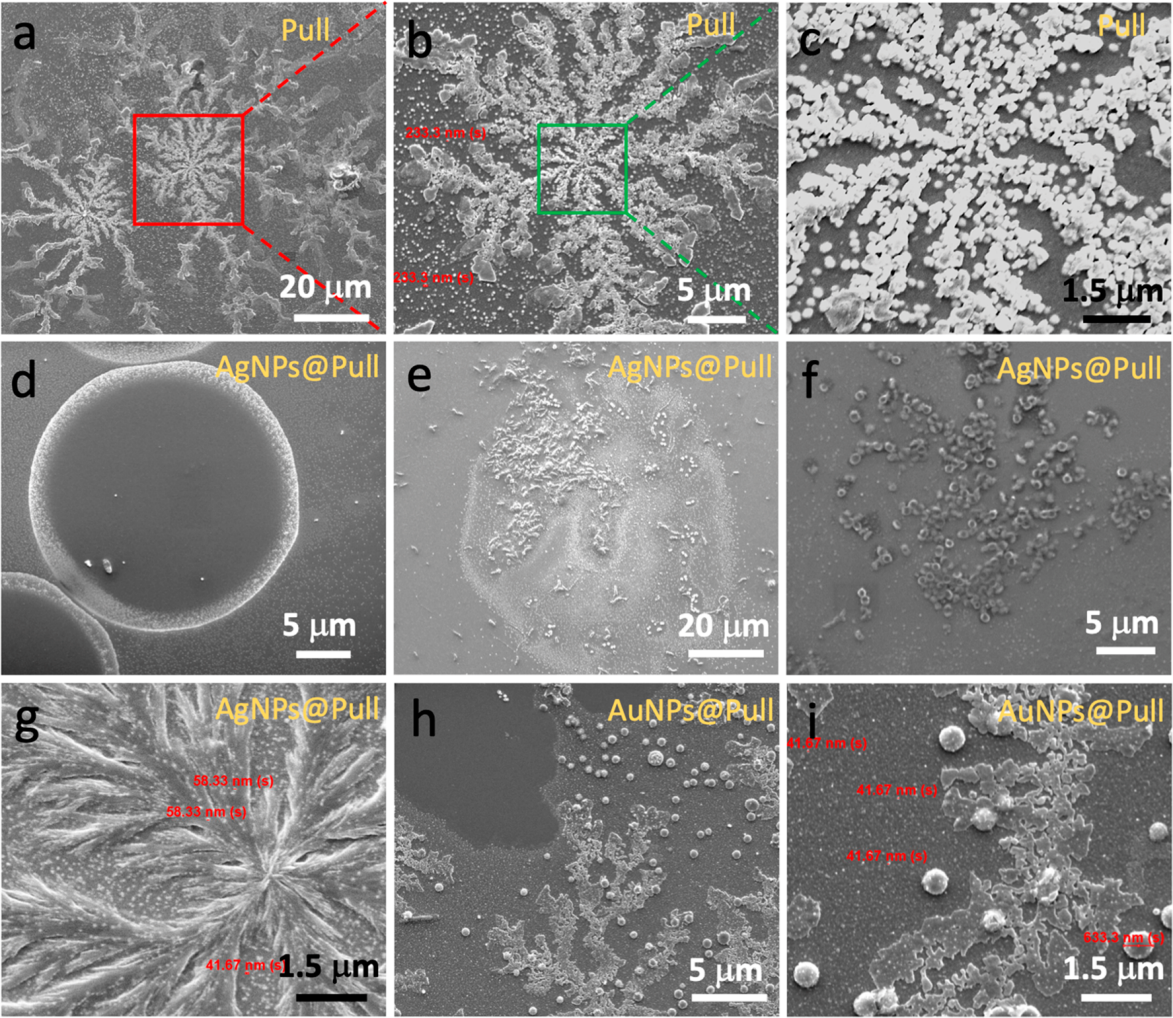
SEM images of microflowerlike self-assembly of nanospheres (∼230 nm) of pristine Pull at magnifications of (a) 2432×, (b) 7500×, and (c) 30 000×. Self-assembly of AgNPs@Pull sample into (d) circular, (e) wormlike, (f) ringlike, and (g) featherlike arrays. SEM images of branchlike self-assembly of spheres (∼630 nm) of AuNPs@Pull at magnifications of (h) 10 000× and (i) 30 000×. All images were taken on the mica surface.

In a single pullulan chain, both α-1,4 and α-1,6 linkages are found: three glucose units connected by α-1,4 glycosidic bonds link to each other by an α-1,6 glycosidic bond.^44^ This alternating linkage pattern is responsible for the high structural flexibility and amorphous organization of pullulan.^45,46^ Because of the flexibility around the α-1,6 linkage and the absence of charged groups, pullulan reportedly tends to coat surfaces smoothly.^47^ Spherulitic formations such as featherlike oriented structures and randomly distributed perfectly round micrometer-scale particles were reported for the first time on pullulan coatings by Farris and co-workers.^48^ The featherlike structures were regarded as dendritic crystals, whereas the spherical particles were considered as semicrystalline self-assemblies originating from thermodynamic incompatibility between pullulan and PET substrate. The incompatibility triggers a phase separation that leads to aggregation of pullulan, yielding a partial crystallization with radial growth around a starting nucleus. Spherical particles already observed in many other natural polymers such as starch,^49^ amylose,^50^ chitosan,^51^ chitin,^52^ and cellulose.^53^ Here, we report for the first time nanoscale and highly oriented spherical pullulan particles as seen in Figure 4. To avoid ambiguity, we will use the term “oriented” to mean a sample in which the pullulan particles and/or fibers are arranged in a certain order. We relate “crystallization” to the development of lateral order, where the molecular chains have zipped up to produce a three-dimensional lattice. In Figure 4a–c, it is clearly seen that pullulan spheres of roughly 200 nm diameter are highly oriented to yield microflowerlike spherulitic formations on mica, a hydrophilic mineral mainly consisting of oxides of Si and Al.^54^ In the present study, we hypothesize that the supramolecular self-assembling behavior of pullulan chains creates these unexpected arrays consisting of condensed pullulan via noncovalent interactions. With regards to supramolecular assembly, it has been reported that hydrophobized polysaccharides such as cholesterol-bearing pullulan yield spherical self-aggregates via hydrophobic interactions as the main driving force.^55−58^

**Figure 5:**
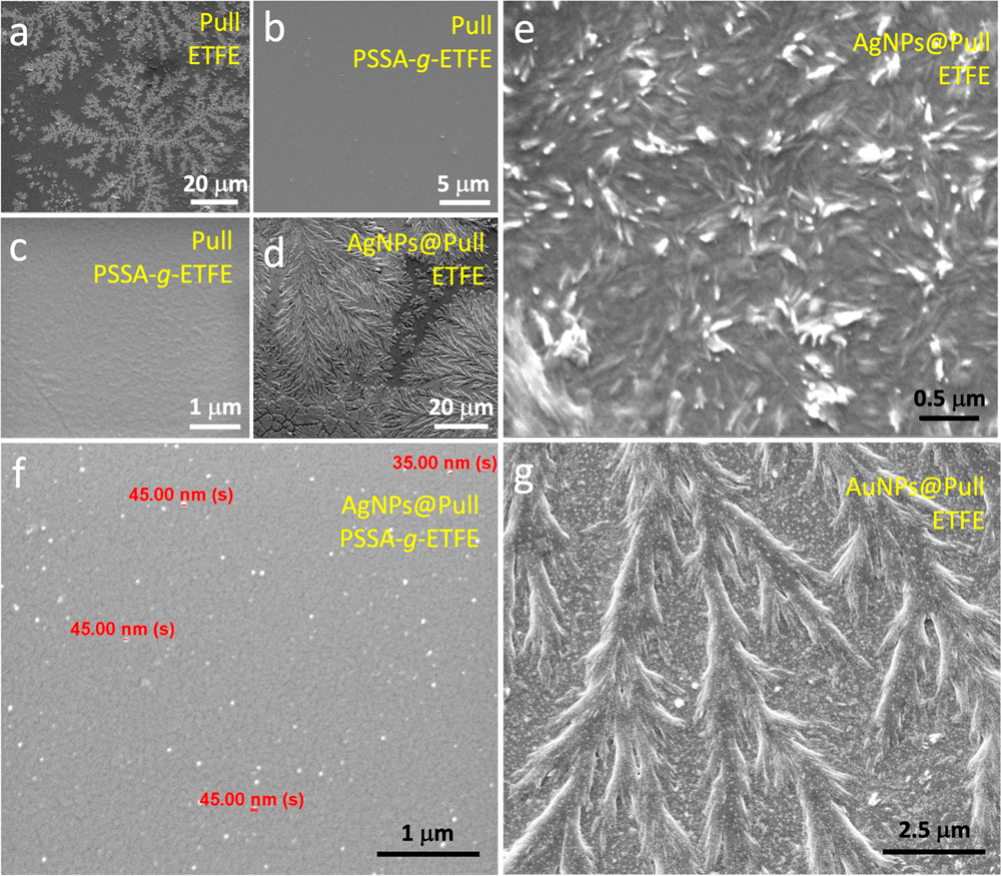
SEM image of (a) Pull on ETFE and (b, c) PSAA-*g*-ETFE film, grafting degree 54%. (d, e) SEM image of AgNPs@Pull on ETFE. (f) SEM image of AgNPs@Pull on PSAA-*g*-ETFE film, grafting degree: 54%. (g) SEM image of AuNPs@Pull on ETFE.

To better understand the self-assembly of pullulan chains, we repeated the SEM analysis on different surfaces. Because mica is already a hydrophilic surface (contact angle, CA: 10°, Figure S6), we first studied on hydrophobic ETFE surface (CA: 93°). SEM analysis performed on the ETFE film showed the formation of nanosized and highly oriented spherical pullulan particles (Figure 5a), similar to those detected on the mica surface. This unexpected result showed us that Pull chains exhibited almost the same behavior on two surfaces of different hydrophobicity. Because neither of these two surfaces have the capacity to make hydrogen bonds, we then performed the SEM analysis on a substrate that has an undeniable hydrogen bonding capacity thanks to the sulfonic acid groups in its structure. SEM analysis carried out on a poly(styrene sulfonic acid)-grafted ETFE film (ETFE-*g*-PSSA, CA: 43°)^19^ showed that Pull chains were completely spread over the surface as a thin layer without forming nanoparticles or microarranged patterns, as can be seen in panels b and c in Figure 5. When the water evaporates, the remaining pullulan chains interact so intensely with each other via hydrogen bonds that they are prevented from spreading to substrates such as ETFE and mica and self-assemble to yield supramolecular arrays. On the other hand, on surfaces that offer strong interactions such as ETFE-*g*-PSSA, pullulan chains spread over the substrate and cover it in the form of a thin film through hydrogen bonds as the main driving force.

The SEM images of AgNPs@Pull deposited on a mica sheet by solvent evaporation at ambient temperature surprisingly indicate that different arrays consisting of circular, wormlike, ringlike, and featherlike arrays are also formed as seen in Figure 4d–g. A closer look (30 000×) at the microarrays of AgNPs@Pull clearly shows the formation of uniformly distributed Ag NPs of about 40–60 nm in size (Figure 4g). The featherlike arrays as in Figure 4g indicate that the crystallinity of Pull increases after the reduction of metal ions, which is consistent with XRD and TGA results. Similar featherlike arrays were observed on the ETFE surface too (Figure 5d). Needlelike pullulan crystallites are clearly visible in a zoomed-in (60 000 ) SEM image, Figure 5e. When the surface was ETFE-*g*-PSSA, AgNPs@Pull was completely spread over the surface in a process mainly driven by hydrogen bonds, similar to pristine pullulan, as can be seen in Figure 5f. This figure also clearly shows Ag NPs with a size of about 40 nm in the pullulan layer covering the surface, in agreement with Figure 4g.

The incorporation of AuNPs in pullulan yields similar supramolecular branchlike spherulitic formations consisting of condensed pullulan on mica as seen in Figure 4h, i. However, the pullulan spheres are significantly bigger (roughly 630 nm) in this case, and these microspheres randomly distributed on the surface. In Figure 4i, gold nanoparticles appearing as dense bright nanodots in the pullulan-coated zone on mica are clearly visible under SEM. The distinct gold nanoparticles of around 40 nm are uniformly distributed throughout the pullulan zone, and even on the surface of perfectly round pullulan microspheres (Figure 4i). AuNPs@Pull in Figure 5g presented featherlike arrays on ETFE, somewhat different from those on mica. This figure also highlights the presence of large numbers of Au NPs among the pullulan microarrays. We speculate that incorporation of Ag and Au NPs into pullulan affects thermodynamics of the interactions which further yields changes in supramolecular associations. The variations in the self-assembling process could possibly be attributed to changes in interactions triggered by H-bonding capacity and increased hydrophobicity as a result of the replacement of −OH functionalities in the pullulan chains with COO^–^ groups after nanoparticle formation. To the best of our knowledge, this is the first time that supramolecular self-assembled arrays of pristine and metal NP-decorated pullulan have been reported. To understand the detailed mechanism of this unique example of supramolecular association, we should study the thermodynamics of the interactions within the pullulan chains and between pullulan, water molecules, and the substrate surface in detail. The data to be obtained could be promising both for understanding of supramolecular assemblies in nature and for the preparation of new materials in biotechnology and medicine.

**Figure 6:**
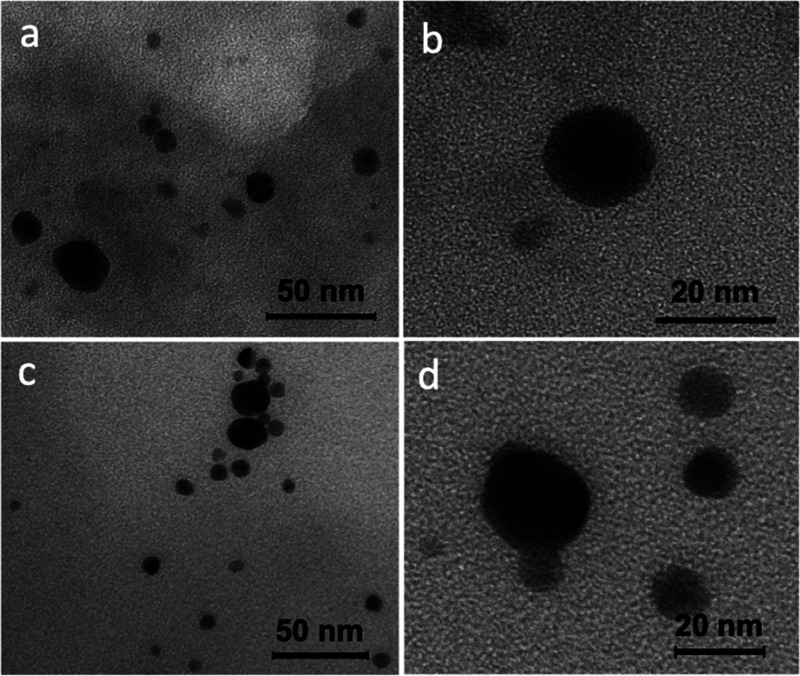
TEM images of (a, b) AgNPs@Pull and (c, d) AuNPs@Pull.

TEM images in Figure 6 showed that metallic Ag and Au nanoparticles of approximately 20–50 nm in size were formed in AgNPs@Pull and AuNPs@Pull, respectively. The images obtained showed that the nanoparticles were uniform; they were all spherical and did not exceed 60 nm in size, and the majority were of the same size.

### 3.3 Bacterial Assays of AgNPs@Pull and AuNPs@Pull

**Figure 7:**
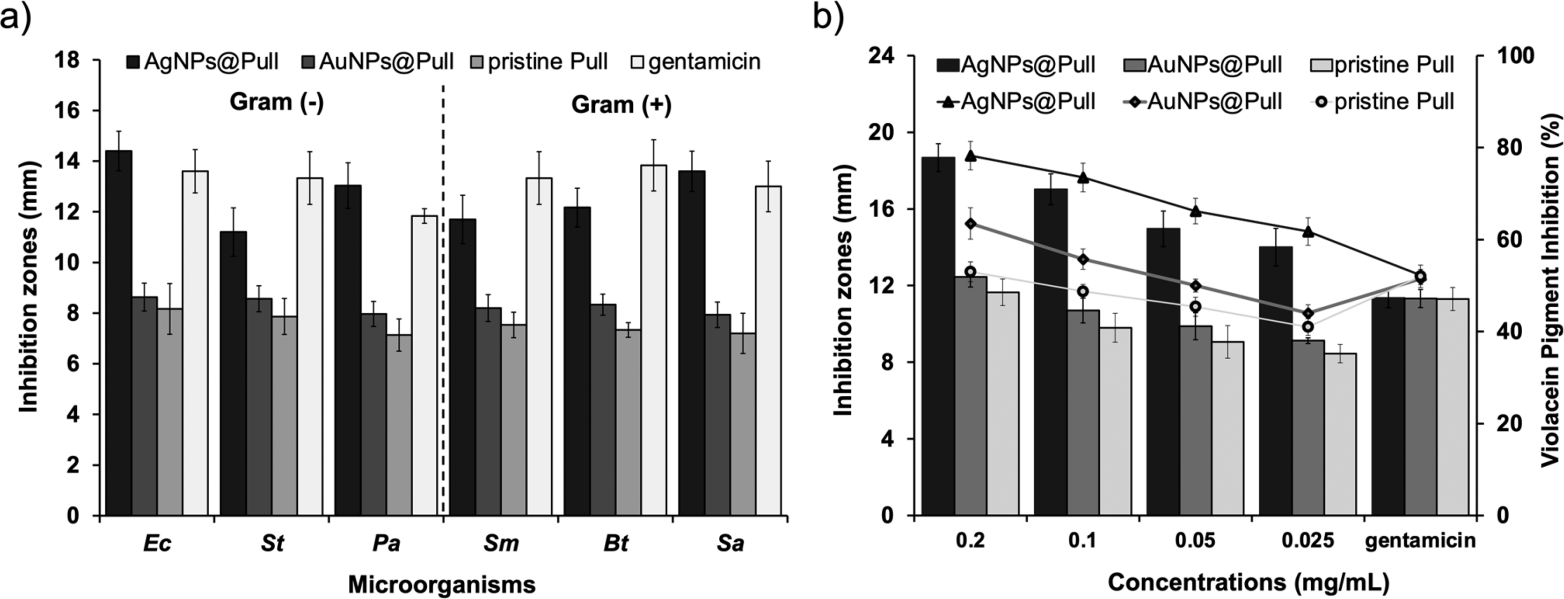
(a) Antimicrobial activities of AgNPs@Pull (0.2 mg/mL), AuNPs@Pull (0.2 mg/mL), pristine Pull (0.2 mg/mL), and gentamicin (10 μg/mL) expressed as inhibition zone diameter (mm). (b) Anti-QS activity of AgNPs@Pull; AuNPs@Pull; pristine Pull with loading levels of 0.2, 0.1, 0.05, and 0.025 mg/mL; and gentamicin (10 μg/mL). Inhibition zone (mm) of AgNPs@Pull (black bars), AuNPs@Pull (dark gray bars) and pristine Pull (light gray bars) and quantitative determination of violacein inhibition (%) for AgNPs@Pull (black triangle), AuNPs@Pull (dark gray square) and pristine Pull (light gray circle).

The antimicrobial effect of AgNPs@Pull and AuNPs@Pull (0.2 mg/mL) was evaluated in comparison with pristine Pull (0.2 mg/mL). The antimicrobial activities of AgNPs@Pull and AuNPs@Pull were tested against human pathogenic common bacteria strains including *Ec*, *Sa*, *Sms*, *St*, *Bt*, and *Pa.* As can be seen in Figure 7a, the antimicrobial activity of AgNPs@Pull and AuNPs@Pull was higher than that of pristine Pull against all bacteria strains, indicating that the antimicrobial effect can be enhanced by the presence of Ag and Au NPs in association with Pull. The highest antimicrobial activity was found against *Ec* as 14.4 ± 0.78 mm and 8.63 ± 0.55 mm for AgNPs@Pull and AuNPs@Pull, respectively. On the other hand AgNPs@Pull and AuNPs@Pull showed the lowest inhibition values against *St* (11.2 ± 0.95 mm) and *Sa* (7.93 ± 0.50 mm), respectively. The inhibition zones of AgNPs@Pull ranged from 14.4 ± 0.78 to 11.2 ± 0.95 mm; these values were substantially larger than the inhibition zones around the discs loaded with AuNPs@Pull, which ranged from 8.63 ± 0.55 to 7.96 ± 0.49 mm. Interestingly, AgNPs@Pull against *Pa* showed a larger zone of inhibition (13.0 ± 0.90 mm) than commercial antibiotic gentamicin (11.83 ± 0.28 mm). Furthermore, the inhibition zones of AgNPs@Pull against *Ec* (14.4 ± 0.78 mm) and *Sa* (13.6 ± 0.79 mm) showed similar inhibition activity as gentamicin (13.6 ± 0.85 mm and 13.1 ± 1.0 mm, respectively). However, AuNPs@Pull and pristine Pull had lower inhibitory values than the commercial antibiotic against all bacterial strains. It was suggested that AgNPs induced by Pull provided an enhanced disruption in the integrity of the bacterial community.^24^ When the inhibition zones of AgNPs@Pull, AuNPs@Pull, and Pull were compared in two bacterial groups, Gram negative (*Ec*, *St*, and *Pa*) and Gram positive (*Sa*, *Sm*, and *Bt*), the values for AgNPs@Pull and AuNPs@Pull were found to be statistically significant in both bacterial groups (*p* < 0.05), whereas the inhibition zones of pristine Pull were not statistically significant (*p* > 0.05) in either group.

To evaluate the effect of loading level of Ag and Au NPs on QS inhibition activity, we administered AgNPs@Pull, AuNPs@Pull, and pristine Pull at four concentrations (0.2, 0.1, 0.05, and 0.025 mg/mL). The QS inhibition activities of AgNPs@Pull, AuNPs@Pull, and pristine Pull were tested against *C. violaceum* CV026 by a disc diffusion assay. Figure 7b shows the effect of AgNPs@Pull, AuNPs@Pull, and pristine Pull in a dose-dependent manner on bioformation of violacein signal molecule (C6-HSL) inhibition by the reporter strain as *C. violaceum* CV026. All concentrations of the studied samples inhibited the violacein signal molecule of *C. violaceum* CV026. Any enhancement in signal molecule inhibition was considered to be related to the increasing loading of AgNPs@Pull, AuNPs@Pull, and Pull. AgNPs@Pull in all loading levels (0.2–0.025 mg/mL) exhibited a higher QS inhibition effect than AuNPs@Pull and pristine Pull according to the radius of clearance zone of violacein pigment around disc. The inhibition zones of AgNPs@Pull (18.67 ± 0.72 mm, 17.025 ± 0.80 mm, 14.96 ± 0.92 mm, and 14 ± 0.98 mm for the loading levels of 0.2, 0.1, 0.05, and 0.025 mg/mL, respectively) were significantly larger than the inhibition zones around the AuNPs@Pull loaded discs (12.45 ± 0.52 mm, 10.7 ± 0.64 mm, 9.88 ± 0.70 mm, and 9.12 ± 0.15 mm for the loading levels of 0.2, 0.1, 0.05, and 0.025 mg/mL, respectively) (Figure S7). The discs containing pristine Pull had the lowest zones of inhibition (ranging from 11.65 ± 0.69 to 8.45 ± 0.47 mm) at each loading level against *C. violaceum* CV026. Interestingly, the commercial antibiotic (gentamicin) showed a lower inhibitory activity (11.35 ± 0.50 mm) than all concentrations of AgNPs@Pull on the violacein pigment production.

The quantitative inhibition of the signal molecule by AgNPs@Pull, AuNPs@Pull, and Pull polymer was determined on the basis of the concentration reduction of the signal molecule against the biomonitor strain as *C. violaceum* ATCC 1247. Similarly, AgNPs@Pull and AuNPs@Pull had a higher effect (ranging from 78.25 ± 3.09 to 61.75 ± 2.98% for AgNPs@Pull and from 63.5 ± 3.41 to 44 ± 1.82% for AuNPs@Pull) on the signal molecule reduction than Pull alone (53.01 ± 2.16–41.02 ± 1.82%) according to absorbance values. AgNPs@Pull inhibited the signal molecule production at the highest level (78.25 ± 3.09) at a loading of 0.2 mg/mL compared to other concentrations. All concentrations of AgNPs@Pull showed a stronger decrease in the signal molecule concentration of *C. violaceum* ATCC 1247 than the commercial antibiotic gentamicin (53 ± 1.25%); however, AuNPs@Pull exhibited a higher signal molecule reduction at loading levels of only 0.2 and 0.1 mg/mL (63.5 ± 3.41% and 55.75 ± 2.21%, respectively) compared to gentamicin (51.5 ± 1.29%). Pristine Pull had almost the same activity (53 ± 2.16%) as gentamicin (52 ± 2.44%) only at a loading level of 0.2 mg/mL (Figure 7b). The use of AgNPs@Pull and AuNPs@Pull as inhibitory agents for bacterial signal molecules has been studied for the first time and, in particular, it has been observed that AgNPs@Pull has the potential to be a therapeutically useful new tool to overcome the bacterial resistance developed against conventional antibiotics.

### Cytotoxicity Assay of AgNPs@Pull and AuNPs@Pull

**Figure 8:**
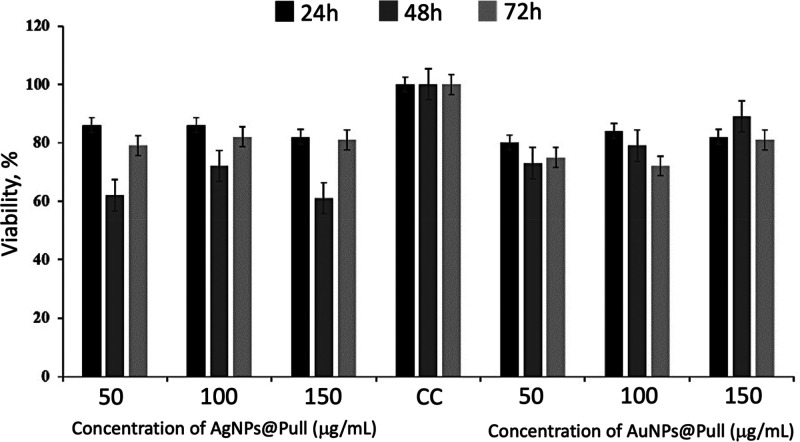
MTT assay results for the in vitro cytotoxicity effects of AgNPs@Pull and AuNPs@Pull against L929 cells for the exposure times of 24, 48, and 72 h. Data are expressed as mean ± SD of six experiments. Percentage of cytotoxicity is expressed relative to untreated controls (*significant *p* < 0.05).

There are limited studies for the cytotoxic effects of biologically synthesized AgNPs@Pull and AuNPs@Pull against cell lines. Therefore, in this study, MTT assay was used to assess the effect of AgNPs@Pull and AuNPs@Pull on L929 cells. The L929 cell line was exposed to various concentrations (50, 100, 150 μg/mL) of AgNPs@Pull and AuNPs@Pull for different periods of time (24, 48, and 72 h) and the cytotoxic activities were found to be statistically significant (*p* = 0.0144 for AgNPs@Pull, *p* = 0.0065 for AuNPs@Pull) and dose dependent (Figure 8). The fibroblasts incubated with AgNPs@Pull and AuNPs@Pull spread well and there was no obvious change in their morphology after 24 h of incubation with NPs, as can be seen in Figure S8. The cytotoxicity of NPs increased with their concentration; the cell viability decreased with increasing NP dose. In addition, AgNPs@Pull had a greater cytotoxic effect compared to AuNPs@Pull even at lower doses.

## 4 Conclusion

In this study, a green and facile route was applied for the synthesis of AgNPs and AuNPs using Pull as a reducing/stabilizing agent, and the resulting constructs (AgNPs@Pull and AuNPs@Pull) were employed in the inhibition of QS. Chemical characterizations of AgNPs@Pull and AuNPs@Pull showed that Ag^+^ and Au^3+^ ions were reduced to yield metallic NPs, whereas the Pull biopolymer was oxidized. FTIR, XPS, ^13^C NMR, and ^1^H NMR spectra verified that the chemical changes occurred. DLS showed that the surface charges of both Ag- and Au-decorated samples were negative, indicating that the ions were reduced to a metallic state. Diffraction peaks obtained by XRD confirmed the FCC structure of crystalline Ag and Au NPs. TGA thermograms showed increased thermal stability after decoration of pullulan with NPs, possibly because of higher chain compactness. Inferring from the SEM images, we believe that the supramolecular self-assembling behavior of Pull chains, driven predominantly by hydrogen bonds, creates unexpected highly oriented microarrays of condensed Pull chains, reported here for the first time. TEM images showed that AgNPs@Pull and AuNPs@Pull were both spherical and about 20–60 nm in size.

The antimicrobial activity of AgNPs@Pull was found to be higher than that of AuNPs@Pull and pristine Pull for all bacterial strains studied. Moreover, the inhibition zone in the presence of AgNPs@Pull was larger compared to gentamicin for *P. aeruginosa*, but quite similar for *E. coli* and *S. aureus*. QS inhibition activity was tested against the CV026 reporter strain (*C. violaceum*) in a dose-dependent manner. Signal molecule inhibition was found to increase with increasing loading level. On the basis of the radius of the clearance zone, the QS inhibition activity of AgNPs@Pull was found to be higher than that of AuNPs@Pull and pristine Pull. Furthermore, gentamicin had a lower inhibition activity than AgNPs@Pull. The MTT assay was used to investigate the effect of AgNPs@Pull and AuNPs@Pull on L929 cells. The cytotoxicity of the synthesized NPs found to be concentration- and time-dependent.

Herein, the employment of AgNPs@Pull and AuNPS@Pull as inhibitory agents against bacterial signal molecules was studied for the first time, and these constructs were shown to be good candidates for this purpose. In particular, given the superior performance of AgNPs@Pull against the commercial antibiotic gentamicin, its potential as a new therapeutic agent that can be used to overcome bacterial resistance to conventional antibiotics is remarkable.

## Supporting Information Available

Medium components used in the production of Pull (Table S1); chart of maximum Pull production (Table S2); XPS survey wide scan spectra (Figure S1); particle size distributions by DLS (Figure S2); ^13^C NMR (Figure S3) and ^1^H NMR (Figure S4) spectra; TGA graphs of Pull, AgNPs@Pull, and AuNPs@Pull (Figure S5); preparation of mica surface and CA measurements on mica, ETFE, and ETFE-*g*-PSSA (Figure S6); Petri plate images (Figure S7); and live cell time-lapse images (Figure S8) (PDF)
