# Supplementary material for: Green and Facile Synthesis of Pullulan-Stabilized Silver and Gold Nanoparticles for the Inhibition of Quorum Sensing
Source: ACS Appl Bio Mater. 2022 Feb 3;5(2):517–27. doi: 10.1021/acsabm.1c00964 (PMC8895461; doi:10.1021/acsabm.1c00964)
Supplement: Supplementary file 1 — mt1c00964_si_001.pdf [file mt1c00964_si_001.pdf]

**Supplementary Information**

**for**

# Green and Facile Synthesis of Pullulan-Stabilized Silver and Gold Nanoparticles for the Inhibition of Quorum Sensing

*Mohammadreza Ghaffarlou[1], Sedef İlk[2,3], Hamideh Hammamchi[4], Feyza Kıraç[1], Meltem Okan[5], Olgun Güven[1], Murat Barsbay[1,*]*

[1]Department of Chemistry, Hacettepe University, Beytepe, 06800 Ankara, Turkey

[2]Faculty of Medicine, Department of Immunology, Niğde Ömer Halisdemir University, 51240 Niğde, Turkey

[3]School of Engineering Sciences in Chemistry, Biotechnology and Health, Department of Chemistry, Division of Glycoscience, KTH Royal Institute of Technology, SE-10691 Stockholm, Sweden

[4]Department of Biology, Hacettepe University, Beytepe, 06800 Ankara, Turkey

[5]Department of Micro and Nanotechnology, Middle East Technical University, Ankara 06800, Turkey

e-mail of the corresponding author: mbarsbay@hacettepe.edu.tr

**Table S1.** Medium components used in the production of Pullulan biopolymer (pH=6.5).

| **Component** | Sucrose | $(NH_4)_2SO_4$ | Yeast extract | $K_2HPO_4$ | $MgSO_4 \cdot 7H_2O$ | NaCl | $MnCl_2$ |
|---|---|---|---|---|---|---|---|
| **g/L** | 50 | 0.6 | 0.4 | 5 | 0.2 | 0.1 | 0.01 |

**Table S2.** The maximum pullulan production by *A. Pullulans* 201253.

| Incubation Conditions | Amount of Polysaccharide Produced (g/L) | Amount Produced Pullulan (g/L) | Resulting Biomass Amount (g/L) |
|---|---|---|---|
| pH 7.5, 28°C, 200 rpm, 5% glucose, 1% yeast extract, 3 days of incubation time | 42.8 | 32.89 | 17.89 |

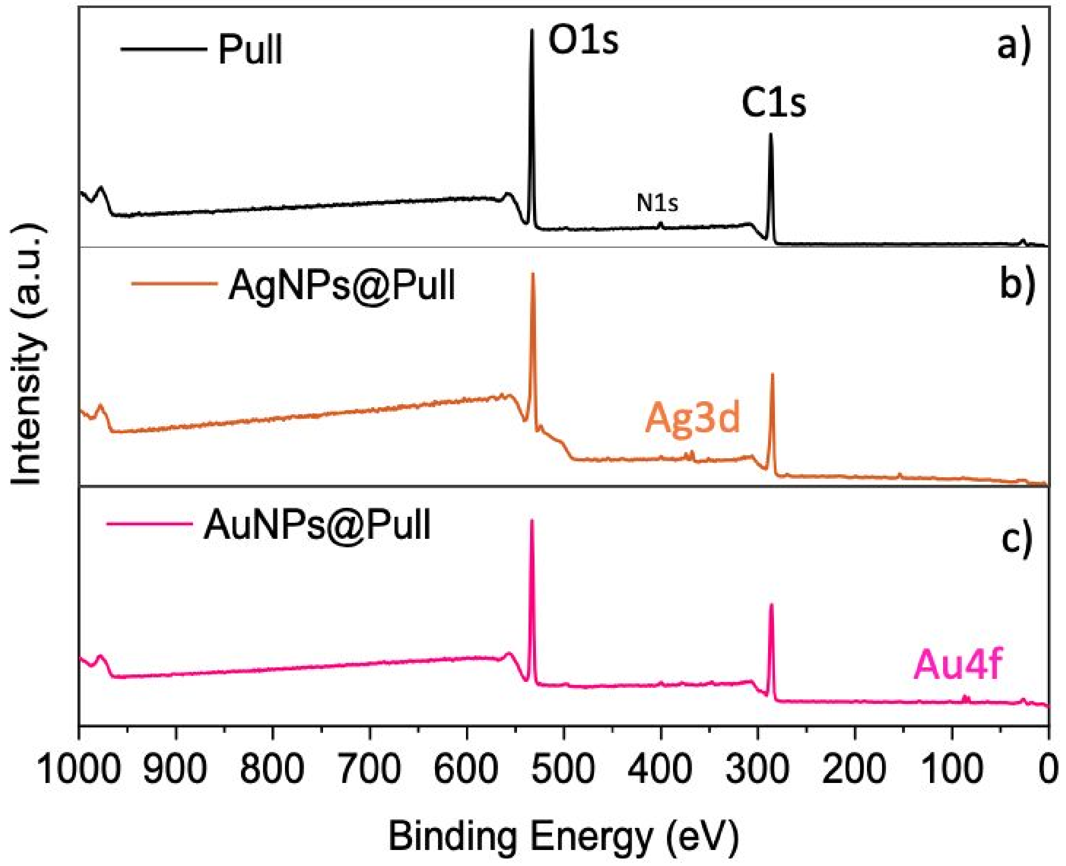


**Figure S1.** XPS survey wide scans of (a) Pull, (b) AgNPs@Pull and (c) AuNPs@Pull.

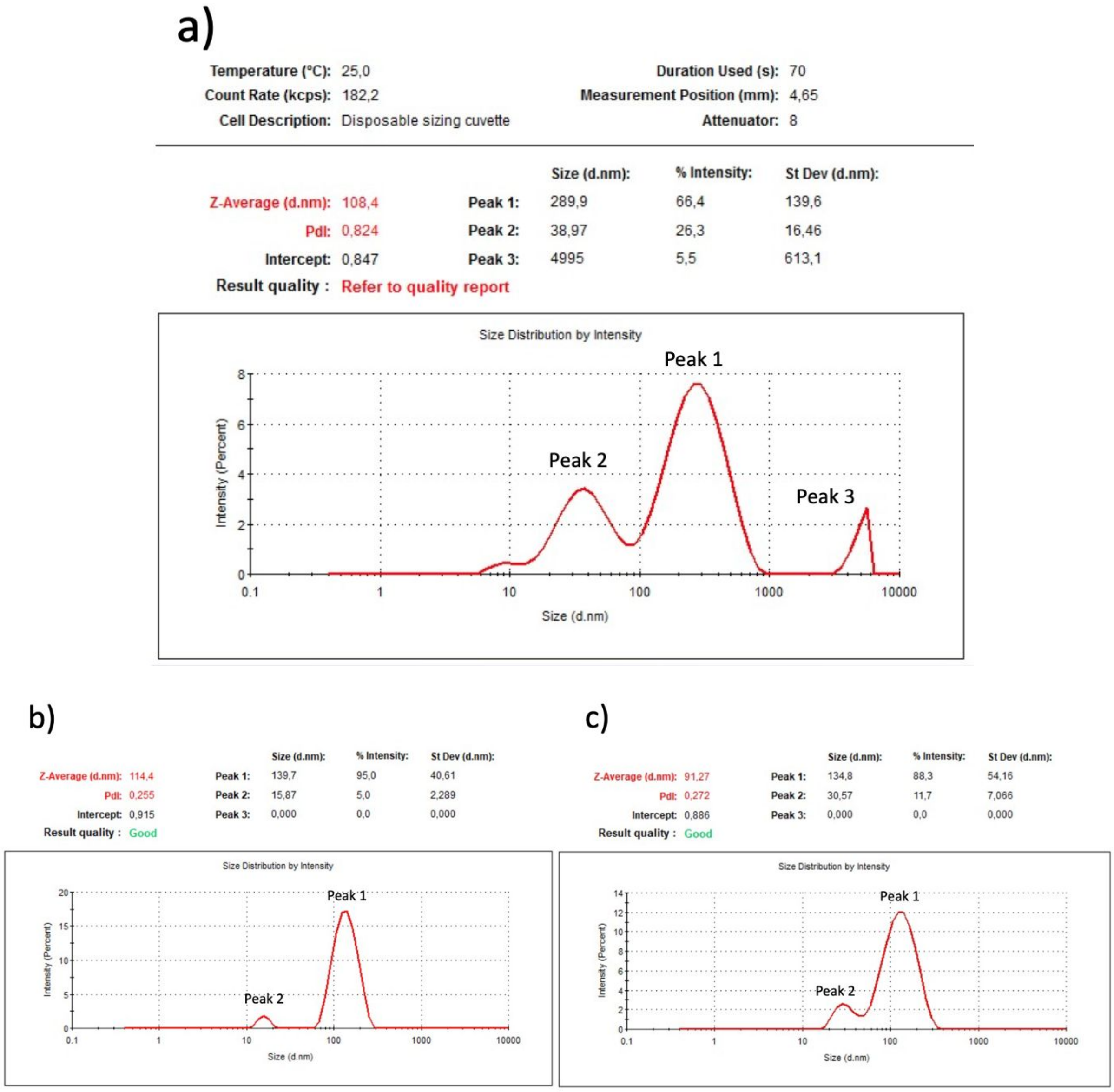


**Figure S2.** Particle size distributions by DLS in 0.1 mg/mL solution in DI water at 25 °C: (a) Pull, (b) AgNPs@Pull and (c) AuNPs@Pull.

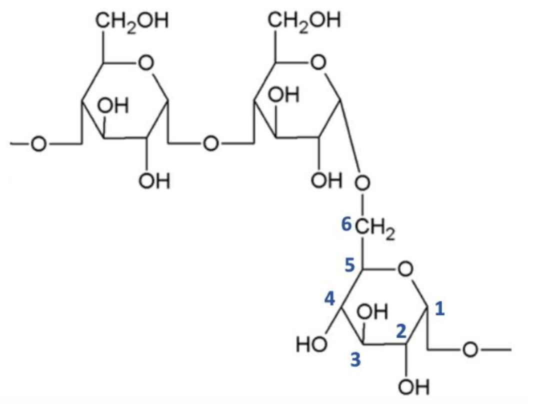

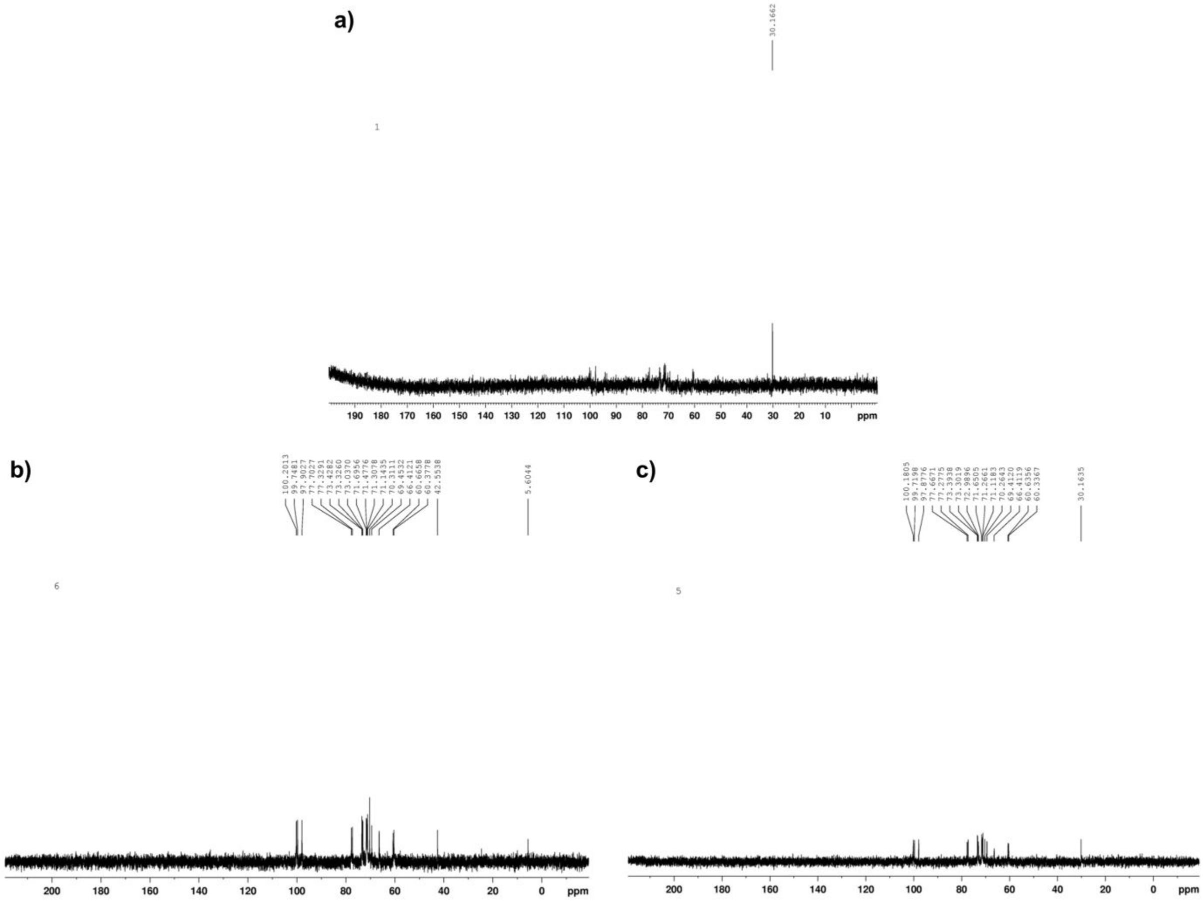


**Figure S3.** $^{13}$C-NMR spectra of (a) Pull, (b) AgNPs@Pull and (c) AuNPs@Pull. The peak at around 31 ppm corresponds to acetone as internal standard.

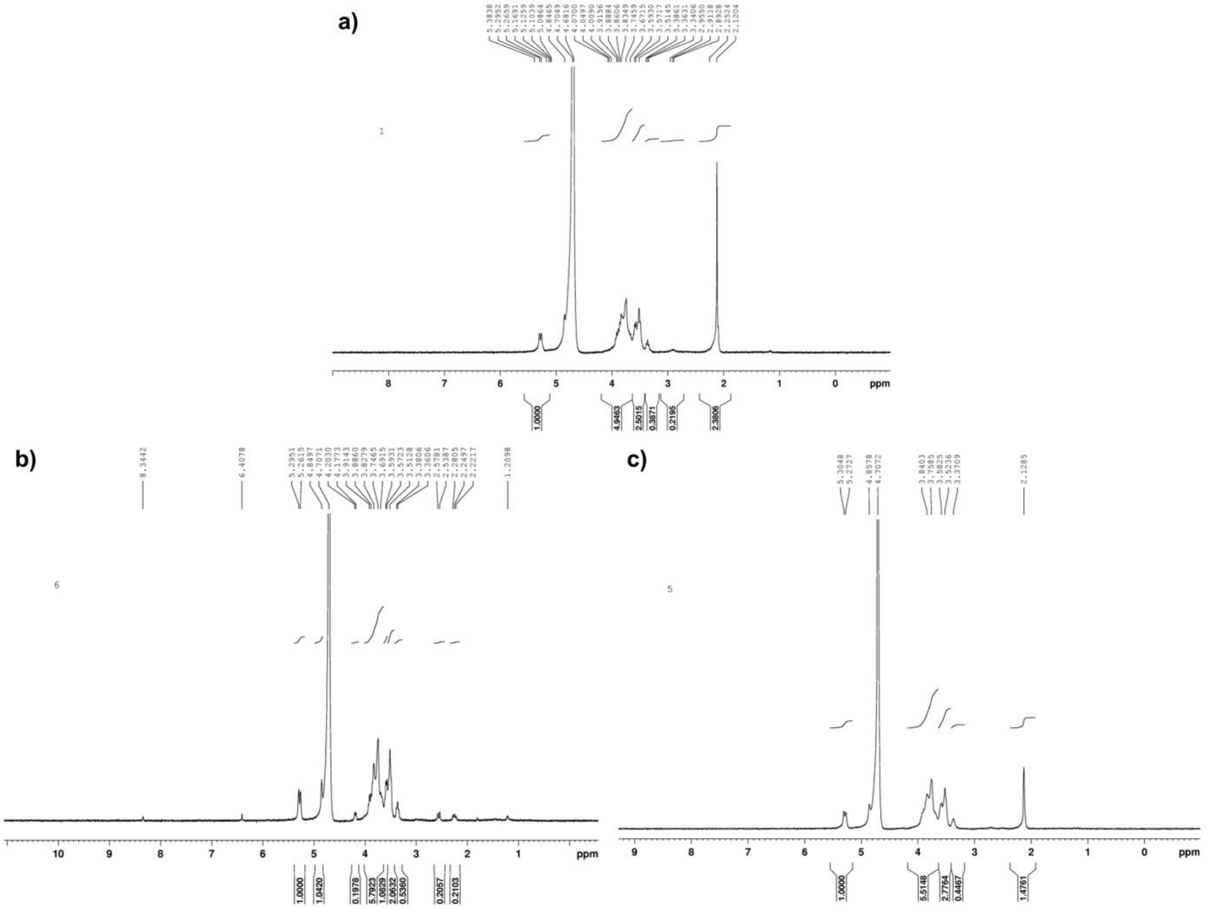

**Figure S4.** $^1$H-NMR spectra of (a) Pull, (b) AgNPs@Pull and (c) AuNPs@Pull. The peak at around 2.2 ppm corresponds to acetone as internal standard.

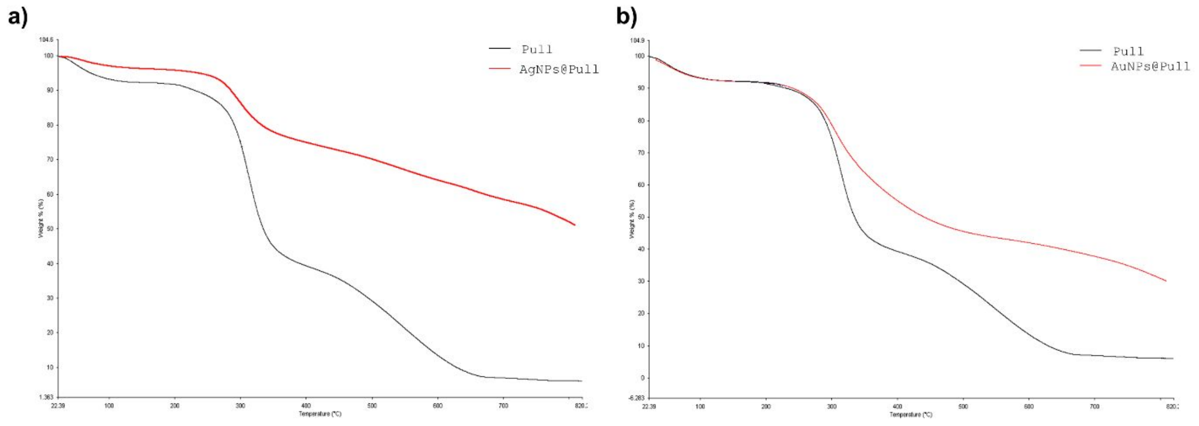


**Figure S5.** TGA graphs of (a) Pull and AgNPs@Pull and (b) Pull and AuNPs@Pull.

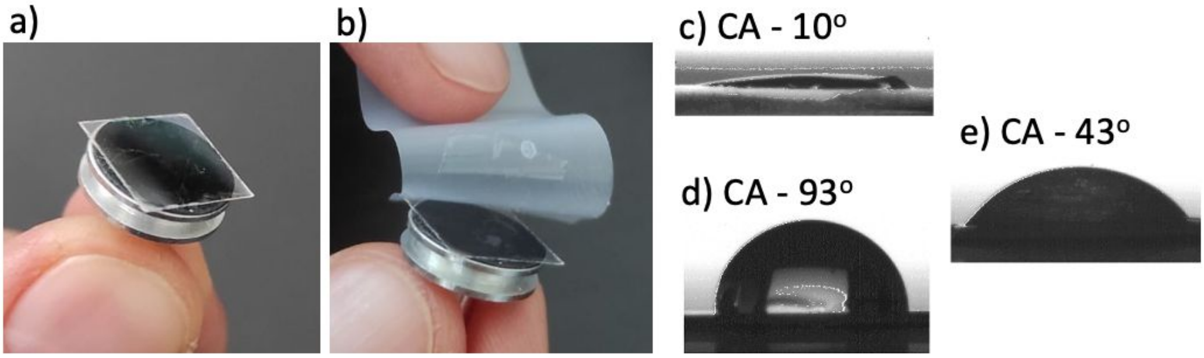


**Figure S6.** (a) Bulk mica surface on an SEM grid, (b) Preparation of fresh/clean mica surface by removing a flake of mica from the surface of bulk mica with the help of an adhesive tape, (c) Contact Angel (CA) of mica surface, (d) CA of ETFE surface, (e) CA of ETFE-*g*-PSSA (grafting degree: 54%).

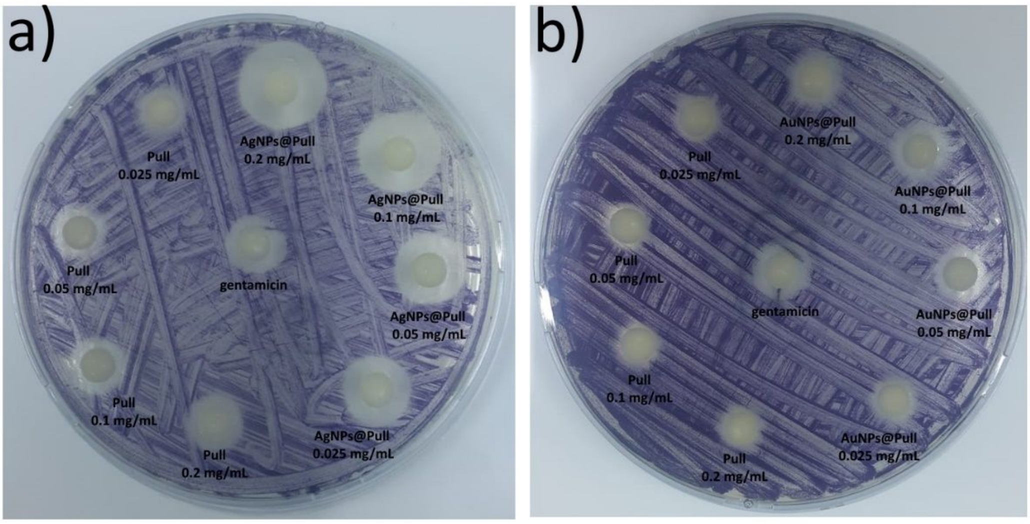


**Figure S7.** Petri plate images of anti-quorum sensing assay for (a) AgNPs@Pull and (b) AuNPs@Pull. Pristine Pull at four different concentrations (0.2, 0.1, 0.05 and 0.025 mg/mL) and commercial antibiotic gentamicin as control were applied along with NPs-decorated Pull (0.2, 0.1, 0.05 and 0.025 mg/mL). The disc diffusion test was carried out by dose dependent manner on bio-formation of violacein signal molecule (C6-HSL) inhibition against reporter strain as *C. violaceum CV026.* A typical photo of the petri plates for each nanoparticle was displayed.

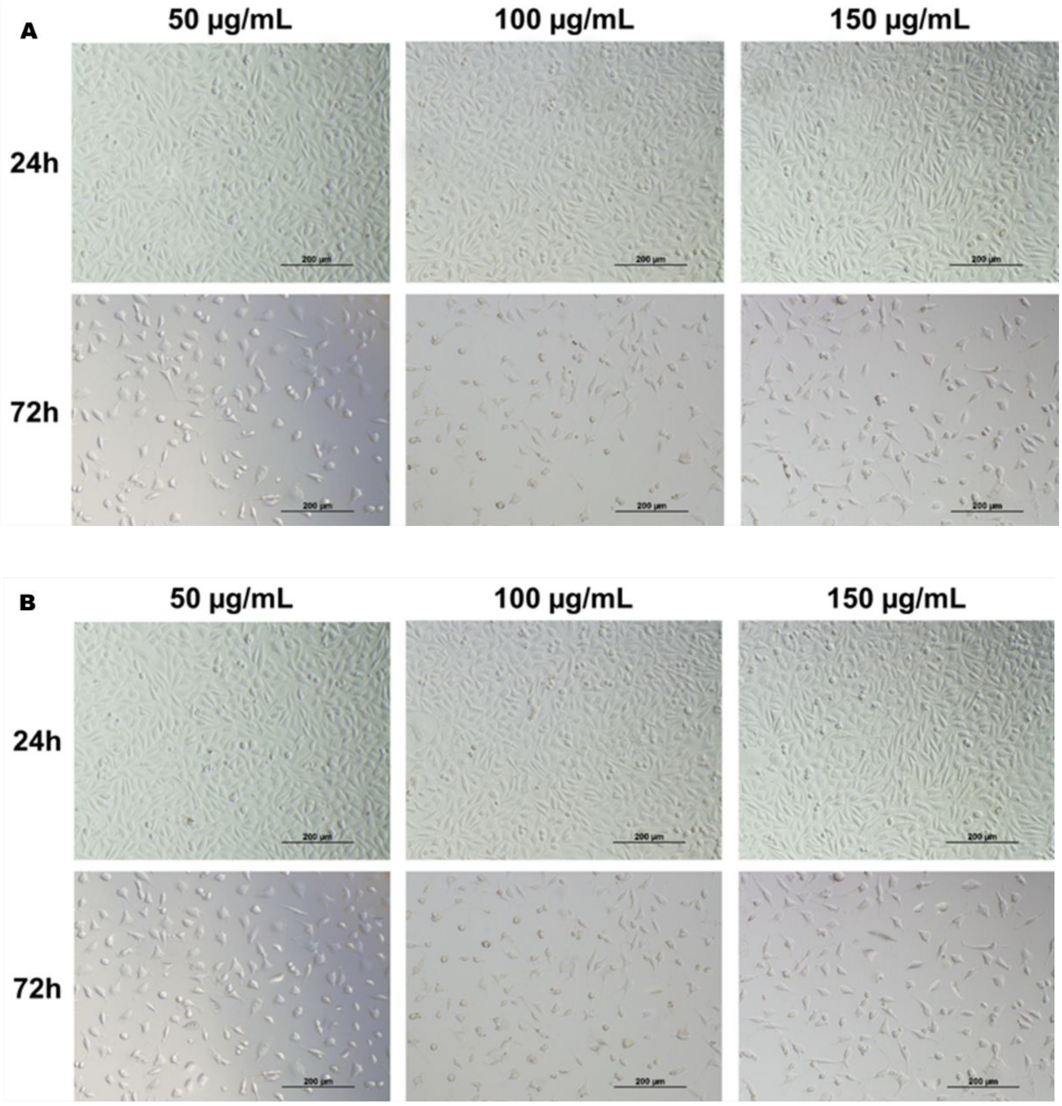


**Figure S8.** Live cell time-lapse imaging of L-929 cells exposed to AgNPs@Pull (A) and AuNPs@Pull (B). Fluorescence microscopy images at 24 and 72 h taken from L-929 cells exposed to 50, 100, 150 μg/mL of 200 nm NPs. Microphotographs were taken with a Leica inverted fluorescent microscope.
